# ALKing the flames of lung cancer immunosensitivity

**DOI:** 10.1002/1878-0261.13533

**Published:** 2023-10-12

**Authors:** Richard Bayliss, Elżbieta Sarnowska, Sharon Yeoh, Josephina Sampson

**Affiliations:** ^1^ School of Molecular and Cellular Biology, Faculty of Biological Sciences University of Leeds UK; ^2^ Department of Experimental Immunotherapy Maria Sklodowska‐Curie National Research Institute of Oncology Warsaw Poland

**Keywords:** cancer vaccine, combination therapy, immunotherapy, lung cancer, tyrosine kinase inhibitors

## Abstract

Immune checkpoint inhibitors (ICIs) are utilised in treating non‐small cell lung cancer (NSCLC) by enhancing the immune response against cancer cells. However, they are not effective against cancers with certain genetic alterations. A recent study by Mota et al. focussed on understanding why ALK+ NSCLC cancers are immune cold and making them more receptive to ICIs using a vaccine‐based approach. The study highlighted cell‐specific differences in the presentation of immunogenic peptides and the location of tumours as factors in the poor immune response. Vaccines based on ALK peptides improved immune response, and when combined with ICIs, this led to a striking improvement in survival in a mouse model of ALK+ NSCLC.

## Graphical abstract

Certain subgroups of non‐small cell lung cancer (NSCLC) patients benefit from immune checkpoint inhibitors (ICIs). A recent study highlighted factors in the poor immune response of ALK+ NSCLC cancers such as cell‐specific differences in the presentation of immunogenic peptides and the tumour site. Vaccines based on ALK peptides combined with ICIs enhanced survival in a mouse model of ALK+ NSCLC.

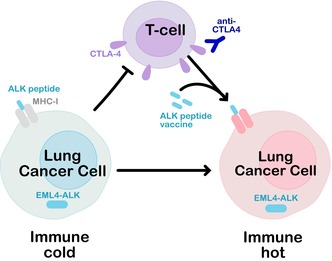

**Abbreviations**
ALCL: anaplastic large‐cell lymphoma
ALK: anaplastic lymphoma kinase
CTLA‐4: cytotoxic T‐lymphocyte‐associated protein 4
EGFR: epidermal growth factor receptor
EML: echinoderm microtubule‐associated protein like
ICI: immune checkpoint inhibitor
NCSLC: non‐small cell lung cancer
NPM: nucleophosmin
PD‐1: programmed death 1
PD‐L1: programmed death 1 ligand
TAPE: tandem atypical propeller domain in EML

Immune checkpoint inhibitors (ICIs) restore the immune‐mediated elimination of cancer cells by blocking the function of proteins such as PD‐1/PD‐L1 or CTLA‐4 that hinder T‐cell proliferation and cytotoxic function. ICIs such as the anti‐PD‐1 antibodies nivolumab and pembrolizumab are standard treatments for advanced non‐small cell lung cancer (NSCLC), showing durable responses and survival improvements in many patients [1]. However, they are not effective in patients who have mutations in *EGFR* or *ALK* rearrangement, approximately 25% of all cases [2]. Most of these patients respond well to tyrosine kinase inhibitors that target these driver mutations, at least initially, but there is a need to improve treatments for patients who do not respond or who relapse [3]. Various explanations have been put forward to explain why ICIs are less effective in these patients compared with other NSCLC patients, such as a tumour microenvironment that excludes or impairs T cells. One potential strategy to boost the immune response to these cancers would be to vaccinate patients with DNA encoding *EGFR* or *ALK*, or peptides taken from the sequences of the proteins they encode. A recent study by Mota et al. [4] piloted a combined vaccine and immunotherapy strategy to target mouse models of ALK+ NSCLC and delved into the mechanisms that underpin immunoresistance in these tumours.

Like human patients, mouse models of ALK+ NSCLC were initially found to be unresponsive to ICIs. However, there was a striking immune response when EML4‐ALK tumours were grown in the flank of the mouse, and tumours in this context were rejected after treatment with ICIs. The poor immune response to ALK is therefore related to the specific location of the tumours, and other recent work suggests that this might be due to poor priming of T cells specifically in lung cancer [5].

Previous work with a DNA vaccine encoding the cytoplasmic domain of ALK produced therapeutic anti‐ALK immune responses in mice [6]. Mota et al. narrowed this down to a set of immunogenic peptides such as a specific 9‐mer sequence from the kinase domain of ALK (hALK1260‐1268) that elicited a strong response from CD8+ cytotoxic T cells in releasing the effector cytokine interferon‐γ. Vaccination of mice significantly increased the number of tumour‐infiltrating T lymphocytes specific for ALK, compared with mice injected with EML4‐ALK tumour cells. This boost to the immune system's recognition had benefit for the survival of ALK+ NSCLC mice treated with the ALK inhibitor lorlatinib, and the addition of an anti‐CTLA4 ICI cured most of the mice treated (Fig. 1).

**Fig. 1:**
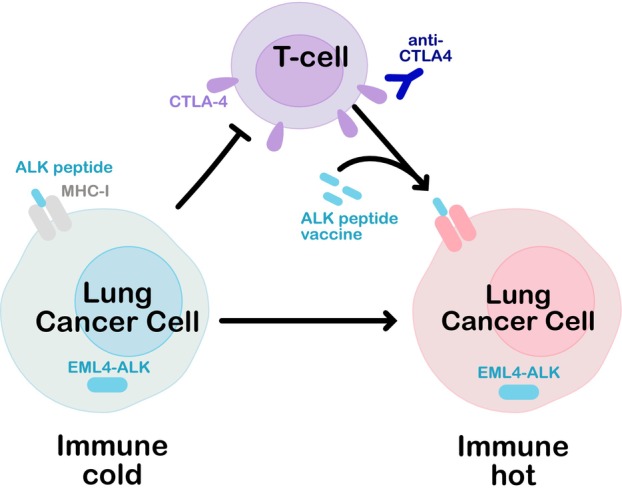
ALK+ NSCLC cancers with certain genetic mutations are immune cold and unresponsive to ICIs. In mouse models, vaccination with immunogenic ALK peptides improves immune response and renders the tumours vulnerable to ICI treatment.

The brain is a major site of metastasis in ALK+ NSCLC, and the development of 2nd‐ and 3rd‐generation ALK inhibitors significantly improved this aspect of therapy because they penetrate the blood–brain barrier. In the paper by Mota et al., the peptide‐based vaccine blocked the formation of new brain metastases, but the effect on already‐established metastases was not tested. ALK is critical in the development of the nervous system but is expressed only in a subset of neurons in adults [7]. However, some patients undergoing ALK inhibitor therapy report cognitive effects that in a few cases require alterations to their dose [8]. The effects of ALK peptide vaccination on the function of the central nervous system were explored by Mota et al. Transgenic mice vaccinated with the ALK peptides showed no evidence of behavioural or other symptoms or of CD8+ T‐cell infiltration into the hypothalamus, a region of the brain enriched in ALK in the mouse. Further studies will be needed to evaluate ALK vaccines with respect to efficacy against brain metastases, and their safety considering the expression of ALK in the human central nervous system.

ALK autoantibodies are found frequently in ALK+ anaplastic large‐cell lymphoma (ALCL) patients, where ALK is fused to the nucleophosmin protein (NPM), and in children a high antibody titre correlated with a lower rate of relapse [9]. ALK autoantibodies have been detected in other ALK+ patients, including NSCLC but at a much lower rate or titre than in ALCL [10, 11]. Mota et al. also observed that ALCL cells present a different selection of the ALK peptides from NSCLC cells. These differences may, at least in part, reflect the underlying levels of ALK fusion protein expression: Whereas NPM‐ALK is expressed highly in ALCL, EML4‐ALK expression levels are relatively low in most patients. Further research is required to probe whether and why the EML4‐ALK fusion in NSCLC cells is less likely to attract the attention of the immune system than the NPM‐ALK fusion in ALCL cells.

Different EML4‐ALK fusion variants arise from distinct breakpoint positions, and the translated proteins vary mostly in the N‐terminal EML4 partner and, most notably, whether a partial TAPE domain is present or absent [12]. All variants have the tyrosine kinase domain of ALK, which is not only the critical biochemical feature for oncogenic signalling but is also the location of immunogenic peptides discovered by Mota et al. All variants of EML4‐ALK would therefore be expected to be recognised by the T cells primed by a vaccine based on ALK kinase domain peptides. However, because this study focussed on the EML4‐ALK V1 model, it is uncertain whether all variants will respond to vaccine and ICIs equally well. This is important because shorter variants, such as V3, relate to a more aggressive and metastatic disease [13]. Other genetic alternations in the cancer cells might be relevant. For example, p53 mutations are correlated with worse patient outcomes in NSCLC, and with modifying the immune environment [13]. Further development of an ALK vaccine necessitates assessing the impact of ALK variant type, p53 status and other markers in connection with stratifying patients and optimal immunotherapy strategy. Resistance to ALK inhibitors may arise through EGFR signalling, and perhaps these cancer cells could also evade immune detection by downregulating EML4‐ALK [14].

Cancer vaccines hold the promise of enhancing the immune system's ability to recognise and target cancer cells, potentially reshaping the field of cancer treatment. This has moved beyond theory for some lung cancer patients as, in Cuba, a vaccine that raises antibodies against EGF (CimaVax‐EGF) is approved for use as a maintenance therapy in advanced NSCLC [15]. The work by Mota et al. contributes to the mounting evidence that ALK is an immunogenic oncoprotein and bolsters the argument that an ALK cancer vaccine is needed to prime the immune system for effective immunotherapy in ALK+ NSCLC patients.

## Conflict of interest

The authors declare no conflict of interest.
